# Meningitis due to *Streptococcus suis* in Two Patients with Occupational Exposure from Northeastern Brazil

**DOI:** 10.1155/2021/5512303

**Published:** 2021-02-24

**Authors:** Francisco Breno Ponte de Matos, Luís Arthur Brasil Gadelha Farias, Camila Negreiro Dias, Lorena Pinho Mendes, Pedro Pinheiro de Negreiros Bessa

**Affiliations:** ^1^Programa de Residência Médica Em Infectologia, Secretária de Saúde Do Estado (SESA), Fortaleza, Ceará, Brazil; ^2^Hospital São José de Doenças Infecciosas (HSJ), Secretaria de Saúde Do Estado Do Ceará, Fortaleza, Ceará, Brazil; ^3^Clínica Médica, Hospital Universitário Walter Cantídeo (HUWC), Fortaleza, Ceará, Brazil

## Abstract

*Streptococcus suis* is an emerging zoonotic agent that causes bacterial meningitis. *S. suis* is an encapsulated Gram-positive, facultative anaerobic bacterium. This infection usually manifests in humans as meningitis, endocarditis, septicemia, and/or arthritis. The primary groups at risk for *S. suis* infection are individuals occupationally exposed to pigs and/or pork, for example, farmers, butchers, and hunters. Herein, we report two cases of meningitis related to *S. suis* with occupational exposure from the Ceará state, northeastern Brazil.

## 1. Introduction

*Streptococcus suis* is an encapsulated Gram-positive, facultative anaerobic bacterium, which may be coccoid or ovoid, and may occur as single cells, in pair, or in chains. It is responsible for major financial losses in the swine industry as it is an emerging zoonotic pathogen with significant public health consequences [1]. Currently, the growing incidence of this pathogen has been described primarily in Asia [1]. This pathogen is found in pigs and colonizes the respiratory and digestive tracts. Pigs have been established as the pathogen's natural reservoir. Transmission to humans may occur during pork product consumption or via occupational exposure [2]. Infection in humans presents clinically as meningitis, endocarditis, septicemia, and/or arthritis, and thus far is primarily reported in Asia [3, 4]. It is rarely observed in western regions, such as Argentina and the United States [5, 6].

Although the pathogen has been reported in pigs from Brazil, there are limited reported cases of infection in humans, specifically in employees in swine slaughterhouses in São Paulo, southeastern Brazil [7]. We report two cases of meningitis related to *Streptococcus suis* from Ceará state, northeastern Brazil, and include a literature review.

Medical records of both patients were reviewed during their respective hospitalizations in March and June 2020 at the Hospital São José de Doenças Infecciosas (HSJ), a tertiary reference infectious disease center. Both patients authorized the publication of their cases and images and provided written informed consent.

## 2. Case 1

A 60-year-old patient who had worked as a butcher in a pig slaughterhouse, who had a history of alcoholism, and who was diagnosed with chronic obstructive pulmonary disease presented to the emergency room with fever (temperature of 38.9°C) and a 1-day history of headache, neck rigidity, motor alteration, and hypoacusis. Physical examination on admission revealed a Glasgow Coma Scale score of 15, nuchal rigidity, and Brudzinski's sign. Kernig's and Lasegue's signs were absent. The remainder of the examination was unremarkable. Laboratory findings on admission revealed a low hemoglobin level (10.5 g/dL) and platelet and white cell counts within the normal range. The patient also presented with abnormal aspartate transaminase (292 IU/L) and alanine transaminase (445 IU/L) levels. Renal function tests were unremarkable.

Based on a suspicion of meningitis, a cerebrospinal fluid (CSF) analysis was performed. This revealed glucose levels of 5 mg/dL, a protein concentration of 143 mg/dL, a white blood cell count of 82 cells/mm³ with 48% polymorphonucleocytes, and a CSF Gram stain with Gram-positive diplococci. Cerebral magnetic resonance imaging revealed nonspecific gliosis and leukoaraiosis. During hospitalization, CSF culture samples were positive for *Streptococcus suis II* that was susceptible to ceftriaxone (MIC, 0.25 *μ*g/mL). Treatment was initiated with ceftriaxone 2 g every 12 h and dexamethasone 4 mg every 6 h. Antibiotic therapy was administered for 14 days, and the dexamethasone was discontinued after 5 days. The patient was discharged with a full clinical resolution of his symptoms. During outpatient follow-up, an audiometry was performed revealing bilateral sensorineural hearing loss.

## 3. Case 2

A 68-year-old farmer with frequent contact with pigs and an uneventful medical history presented to the emergency department with a 3-day history of fever (temperature of 39°C), occipital headache, asthenia, drowsiness, and dysarthria. His clinical course deteriorated the following day with disorientation and symptoms of an altered state of consciousness. On physical examination, his Glasgow Coma Scale score was 10 (verbal response scored 0). He was disoriented for time and space and displayed mutism and nuchal rigidity.

Computed tomography of the brain revealed normal findings. A CSF analysis showed glucose levels of 22 mg/dL, a protein concentration of 320 mg/dL, a cell count of 1,322 cells/mm^3^ with 44% polymorphonucleocytes, and a Gram stain with no findings. CSF culture samples revealed *S. suis I* susceptible to piperacillin-tazobactam (MIC, 0.06 *µ*g/dL). Treatment was initiated with piperacillin-tazobactam 4.5 g every 6 h for 7 days. Due to secondary pulmonary involvement and positive blood samples for *Pseudomonas aeruginosa*, his treatment was changed to meropenem 1 g every 8 h for 14 days and was completed with polymyxin B 25.000 UI per kg per day for 7 days. Administration of corticosteroid and antiparasitic drugs was also included in the treatment. The patient's clinical symptoms improved, and he was discharged after 28 days. Currently, he receives outpatient follow-up care and has exhibited no evident sequelae.

Clinical profile of both patients diagnosed with *Streptococcus suis* meningitis is given in Table 1.

## 4. Discussion

To the best of our knowledge, our case report is the first description of meningitis caused by *Streptococcus suis* from northeastern Brazil in the medical literature. A meta-analysis performed by van Samkar et al. including a total of 913 patients showed that male sex (581/711, 82%), occupational exposure to pigs and raw pig products (395/648, 61%), and alcoholism (60/322, 19%) were important predisposing factors [4]. Our case study shows occupational exposure as a predisposing factor. There was no history of skin injury during direct contact with pigs or raw pig products. We hypothesized that skin injury occurred but was undiscovered or underestimated by the patients. The same meta-analysis also described the primary clinical symptoms: headache (429/451, 95%), fever (514/528, 97%), neck rigidity (462/496, 93%), and an altered state of consciousness (35/113, 31%). The classic meningitis triad is characterized by headache, fever, and an altered state of consciousness (4/43, 9%) [4]. In the cases described in this report, the classic triad was presented in the second case.

Both patients had positive cultures for *Streptococcus suis* in our case study. In contrast, data from the meta-analysis of 913 patients reported that CSF cultures were positive in 758 patients (83%, 95% confidence interval 81–85%) [4]. In a report by Suankratay et al., a pathogen was isolated from the CSF in 66.7% of patients and in blood from 50% of the patients [8]. In our report, although CSF cultures were positive, blood cultures were negative in both cases.

Case 1 presented hearing loss as a sequela to meningitis. Therefore, an otorhinolaryngology consult and an audiometry are essential for patients with *S. suis* meningitis early in their clinical course. It is believed that *S. suis* can directly affect the auditory nerve or cause hemorrhagic labyrinthitis [9, 10]. A risk scoring system for predicting *S. suis* hearing loss is under development [11]. Despite considerable prevalence, little is known regarding the etiopathogenesis of hearing impairment observed in the later stages of *S. suis* meningitis.

An additional significant finding was the increase of transaminases in Case 1. This can indicate systemic involvement, mainly liver involvement due to bacteremia. This was reversible during the medical follow-up.

The present article describes two cases of *S. suis* meningitis from Ceará state, northeastern Brazil. In Brazil, *S. suis* has previously been identified in swine from 13 states: Rio de Janeiro, São Paulo, Minas Gerais, Paraná, Santa Catarina, Bahia, Mato Grosso, Mato Grosso do Sul, Rio Grande do Sul, Pernambuco, Distrito Federal, Espírito Santo, and Goiás. This demonstrates the critical nature of this pathogen in Brazil [12]. The first published well-described case of meningitis related to *S. suis* was in an elderly patient from Rio de Janeiro [13]. This is a remarkable fact that emphasizes the emergence of this pathogen and its clinical importance.

The majority of countries have reported initial cases of *S. suis* infection [13–15]. Although *S. suis* seems to be a concern primarily in countries with a high incidence of *S. suis* infections, it should not be neglected elsewhere. This is especially true after a study conducted in New Zealand showed a high ratio of local farmers and meat inspectors that were seropositive [16]. Similar studies have been published in Brazil focusing on butchers and the swine industry [12].

In summary, *S. suis* infection is an emergent zoonosis with significant sequelae that can lead to death. Patients presenting with meningitis should be tested for *S. suis*, especially if they report a risk of exposure. Raising awareness among clinicians and providing appropriate education for people handling pigs or raw pig products are essential measures for the prevention and control of this newly emerging zoonosis. We hope that this commentary will prompt the reporting of similar cases in Brazil.

## Figures and Tables

**Table 1:** Clinical profile of both patients diagnosed with *Streptococcus suis* meningitis.

Demographic characteristics				Clinical findings					Treatment	
Patient	Age	Exposure	Comorbidities	Fever	Neck stiffness	Brudzinski	Kernig	Lasegue	Antibiotic	Corticosteroids
Case 1	60 y	Butcher (pig slaughterhouse)	COPD/alcoholism	Yes	Yes	Yes	No	No	Ceftriaxone (14 days)	Yes
Case 2	68 y	Farmer (pig farming)	None	Yes	Yes	No	No	No	Piperacillin-tazobactum (7 days) Meropenem (14 days) Polymyxin B (7 days)	Yes
_CSF analysis								Prognosis		
Patient	Appearance	Glucose (mg/dL)	Protein (mg/dL)	Gram stain	WCC (cells/mm^3^)	Cell differential	Method	Outcome		
Case 1	Turbid	5	143	Gram-positive diplococcus	82	PMN (48%), L (42%), and others (10%)	Culture, *Streptococcus suis II*	Discharge		
Case 2	Normal	22	320	No findings	1,322	PMN (44%), L (52%), and others (4%)	Culture, *Streptococcus suis I*	Discharge		

y, years; COPD, chronic pulmonary obstructive disease; CSF, cerebrospinal fluid; WCC, white cell count; PMN, polymorphonuclear cells; L, lymphocytes.

## Data Availability

The data used to support the findings of this study are available from the corresponding author upon request.
